# Cancer: A bioelectric disease?

**DOI:** 10.1002/ctm2.70701

**Published:** 2026-05-21

**Authors:** Celine Desoyer, Roko Šupe, Sahar Ghorbanpour, Debkalpa Goswami, Mina Khalaj, Nazanin Karami, Dagmar Brislinger, Charlotte A. E. Hauser, Christian Baumgartner

**Affiliations:** ^1^ Institute of Health Care Engineering with European Testing Center of Medical Devices Graz University of Technology Graz Austria; ^2^ Department of Cell Biology, Histology and Embryology, Gottfried Schatz Research Center Medical University of Graz Graz Austria; ^3^ BioTechMed Graz Graz Austria; ^4^ Laboratory for Nanomedicine, Division of Biological and Environmental Science and Engineering (BESE) and Computational Bioscience Research Center (CBRC) King Abdullah University of Science and Technology Thuwal Kingdom of Saudi Arabia

**Keywords:** bioelectric biomarkers, bioelectric signalling, cancer bioelectricity, ion channels in cancer, membrane potential (*V*_m_), precision oncology, tumour progression and stemness, tumour‐treating fields

## Abstract

Cancer progression is driven by coordinated alterations in signalling networks that regulate proliferation, plasticity, metabolism and therapeutic response. Although genetic and epigenetic mechanisms are well characterised, there is an increasing body of evidence that suggests bioelectric signalling constitutes an additional integrative regulatory layer in tumour biology. In diverse experimental systems, malignant cells consistently exhibit depolarised transmembrane potentials (*V*_m_), which correlate with proliferation, stemness, invasion and therapy resistance, suggesting depolarisation as a conserved bioelectric hallmark of malignancy. This shifts the central question from whether cancer can be considered a bioelectric disease to the extent to which bioelectric signalling constitutes a relevant organising dimension of tumour biology.

However, a quantitative, translationally actionable framework for membrane potential in cancer is lacking. Existing studies and reviews have largely focused on individual ion channels, specific tumour contexts, or conceptual aspects of bioelectricity without systematically establishing *V*_m_ as a cross‐tumour, systems‐level state variable.

Here, we summarise approximately 15 years of experimental and translational research to evaluate the extent to which *V*_m_ functions as an integrative regulatory dimension of malignancy. Here, we define a state variable as a measurable, dynamically tuneable parameter that integrates multiple regulatory inputs and predicts system‐level cellular behaviour.

At the same time, we identify key limitations in the current evidence base, including limited quantitative comparability across tumour types, incomplete mechanistic integration across regulatory layers, insufficient resolution of tumour heterogeneity and a lack of standardisation for clinical translation.

Based on this review, we introduce a quantitative framework and a structured translational roadmap for incorporating bioelectric state control into precision oncology. This establishes membrane potential as not only a supplementary biomarker, but also a functional pharmacodynamic indicator and an actionable control variable for state‐guided therapeutic intervention.

## Highlights

- Membrane potential (*V*_m_) emerges as a quantitative, systems‐level state variable that integrates ion‐channel activity with signalling, metabolism and cell‐state control across tumour types.
- Across cancer models, depolarised *V*_m_ is a conserved bioelectric hallmark of malignancy, with context‐dependent heterogeneity reflecting tumour type, stage and microenvironment.
- *V*_m_ dynamics can precede phenotypic transformation and actively regulate proliferation, stemness, migration and response to therapy.
- As a measurable and tuneable parameter, *V*_m_ can serve as a functional biomarker and pharmacodynamic readout, facilitating bioelectric‐guided stratification and intervention.
- A structured translational roadmap positions *V*_m_ as an actionable control variable and therapeutic entry point within precision oncology frameworks.

## Graphical abstract

The membrane potential (*V*_m_) has emerged as a systems‐level regulator of cancer cell behaviour, linking ion channel activity to processes such as proliferation, differentiation and therapeutic response. This framework provides a roadmap for translating *V*_m_ measurement and validation into bioelectric precision medicine, integrating multi‐omics and computational modelling. Ultimately, *V*_m_‐guided diagnostics and adaptive interventions could facilitate the real‐time monitoring and control of tumour states, reimagining cancer as a disorder of dysregulated bioelectric signalling.

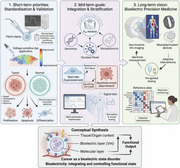

## 1 BACKGROUND AND CONCEPTUAL FRAMEWORK

The transmembrane potential (*V*_m_), also referred to as steady‐state membrane voltage, is generated by asymmetric ionic gradients and selective membrane conductance. Although it was initially studied in excitable tissues, *V*_m_ is now recognised as a ubiquitous bioelectric state variable that influences and coordinates fundamental cellular processes, such as proliferation, differentiation, migration, and long‐range cell‐to‐cell communication, in non‐excitable tissues.^1^, ^2^, ^3^ In cancer, accumulating evidence suggests that bioelectric dysregulation acts as both a downstream consequence of oncogenic signalling and, in certain contexts, an upstream regulator of malignant behaviour.^2^, ^3^, ^4^, ^5^

Cancer cells frequently exhibit depolarised membrane potentials relative to matched non‐transformed cells across tumour types.^6^, ^7^, ^8^, ^9^, ^10^, ^11^ Experimental manipulation of *V*_m_ can alter proliferation, invasion, differentiation, and phenotypic stability. In selected systems, it can also partially decouple malignant behaviour from specific genetic backgrounds.^3^, ^4^, ^10^, ^12^, ^13^, ^14^, ^15^, ^16^ These observations suggest that *V*_m_ extends beyond a descriptive correlate of transformation and represents a reversible, drug‐modulable control variable with diagnostic and therapeutic relevance.^13^, ^17^, ^18^

The idea that bioelectric properties influence proliferation and oncogenic transformation is not new. Early work by H. S. Burr proposed that endogenous bioelectric fields are associated with cancer development, suggesting that alterations in electrical properties may precede morphological changes. This concept was later refined by studies demonstrating that sustained depolarisation accompanies mitotic entry and malignant transformation, whereas hyperpolarisation is associated with growth arrest and differentiation.^19^, ^20^, ^21^ Advances in electrophysiology, calibrated voltage imaging and computational modelling now allow us to more precisely investigate whether *V*_m_ functions as an upstream regulator, a permissive state variable or a downstream readout of oncogenic processes.^2^, ^22^, ^23^, ^24^, ^25^ Therefore, an important question is not whether cancer can be considered a bioelectric disease, but to what extent bioelectric signalling constitutes an organising dimension of tumour biology. Addressing this requires expanding channel‐centric perspectives towards a quantitative, systems‐level understanding of membrane potential as an integrative state variable.

Despite this progress, three key gaps remain. Firstly, quantitative comparisons of resting membrane potentials across tumour types are limited. Secondly, most studies focus on individual ion channels or specific tumour contexts, while *V*_m_ as an integrated, systems‐level variable remains insufficiently addressed. Thirdly, the translational potential of bioelectric state control has not yet been systematically incorporated into precision oncology frameworks. This review addresses these gaps by combining a quantitative synthesis of *V*_m_ across cancer systems with a mechanistic, systems‐level framework and a structured, translational roadmap.

### 1.1 The membrane potential as a systems‐level regulator

The membrane potential is a fundamental biophysical property that is required for cellular homeostasis, transport processes, and signalling. Many electrogenic transport mechanisms exploit the membrane potential directly as a source of free energy, thereby linking ionic gradients to nutrient uptake and metabolic regulation.^26^ At steady state, *V*_m_ can be approximated by the Goldman–Hodgkin–Katz equation, which reflects the relative permeabilities and concentrations of major ions.^27^ However, in proliferative and malignant cells, *V*_m_ is inherently dynamic and cannot be fully captured by equilibrium descriptions.

Importantly, *V*_m_ is not determined by a single ionic species, but emerges from the integrated activity of ion channels, transporters, and electrogenic pumps. While potassium conductances often dominate in differentiated, non‐proliferative cells, sodium, calcium and chloride fluxes, together with transporters such as the Na^+^/K^+^‐ATPase and sodium‐coupled nutrient transporters, substantially contribute to *V*_m_ regulation in proliferative and cancer‐associated states.^23^, ^28^, ^29^, ^30^ *V*_m_ should therefore be understood as an emergent, systems‐level property integrating membrane biophysics with signalling, metabolism, and cell‐state regulation.^1^, ^31^, ^32^, ^33^, ^34^

### 1.2 *V*_m_ in proliferation and cellstate control

Proliferative capacity is closely associated with membrane potential across tissues. Non‐proliferative cells typically exhibit hyperpolarised values of *V*_m_, whereas actively dividing and malignant cells operate in more depolarised regimes.^3^, ^7^, ^8^, ^9^, ^10^, ^20^, ^23^, ^28^, ^30^, ^35^ However, this distinction is not static, but is instead dynamically regulated during the cell cycle. Quiescent cells are generally hyperpolarised; depolarisation accompanies entry into the G1 phase; hyperpolarisation supports DNA synthesis in the S phase; and further depolarisation occurs during the G2/M transition.^20^, ^22^, ^25^

These voltage transitions are not merely correlative.^20^, ^22^ Membrane depolarisation can reorganise charged phospholipids, thereby facilitating K‐Ras nanoclustering and the activation of MAPK signalling pathways.^36^ Conversely, experimentally induced hyperpolarisation can arrest proliferation reversibly by blocking DNA synthesis and mitotic entry.^10^, ^13^ These findings support a model in which *V*_m_ functions as a gating variable for cell‐cycle progression.^34^

Over the past decade, a variety of voltage‐to‐biochemistry transduction mechanisms have been identified, including voltage‐dependent regulation of lipid organisation, ion flux‐mediated second messenger signalling (e.g. Ca^2^^+^ and pH) and electrostatic control of membrane‐associated signalling complexes. These mechanisms collectively link *V*_m_ changes to downstream cellular behaviours.^23^, ^29^, ^36^

At a mechanistic level, these states arise from coordinated changes in ion‐channel activity, transporter flux, and pump–leak balance. Non‐proliferative cells are characterised by dominant potassium conductances and robust Na^+^/K^+^‐ATPase activity, which maintain hyperpolarised states.^22^, ^28^, ^37^ In contrast, proliferative and malignant cells exhibit increased Na^+^ influx, altered Ca^2^^+^ and Cl^−^ fluxes, and engagement of electrogenic transporters, thereby stabilising depolarised *V*_m_ states.^13^, ^23^, ^24^ This ensemble‐based remodelling links membrane potential directly to proliferation control.

### 1.3 Bioelectric mechanisms in cancer: channels, transporters and networks

The membrane potential and ion transport are tightly coupled. *V*_m_ emerges from the integrated activity of ion channels, transporters and pumps, while simultaneously being regulated by them through the transmembrane electric field.^28^ In cancer, this coupling is systematically altered through the coordinated remodelling of ion transport systems, which is often referred to as ‘oncochannelopathies’.^23^, ^24^, ^38^

Voltage‐gated sodium channels, including Nav1.5 and Nav1.7, are frequently overexpressed in multiple cancers, contributing to depolarisation, migration and invasion.^39^, ^40^, ^41^, ^42^, ^43^, ^44^, ^45^ Potassium channels such as Kv1.3, Kv1.5 and Kv11.1 (hERG) modulate resting *V*_m_ and influence proliferation and apoptosis in a context‐dependent manner.^22^, ^46^ Calcium and chloride channels extend these effects into intracellular signalling networks by linking *V*_m_ to Ca^2^^+^‐dependent pathways, cytoskeletal dynamics, and volume regulation.^23^, ^29^, ^33^, ^47^, ^48^, ^49^

**FIGURE 1:**
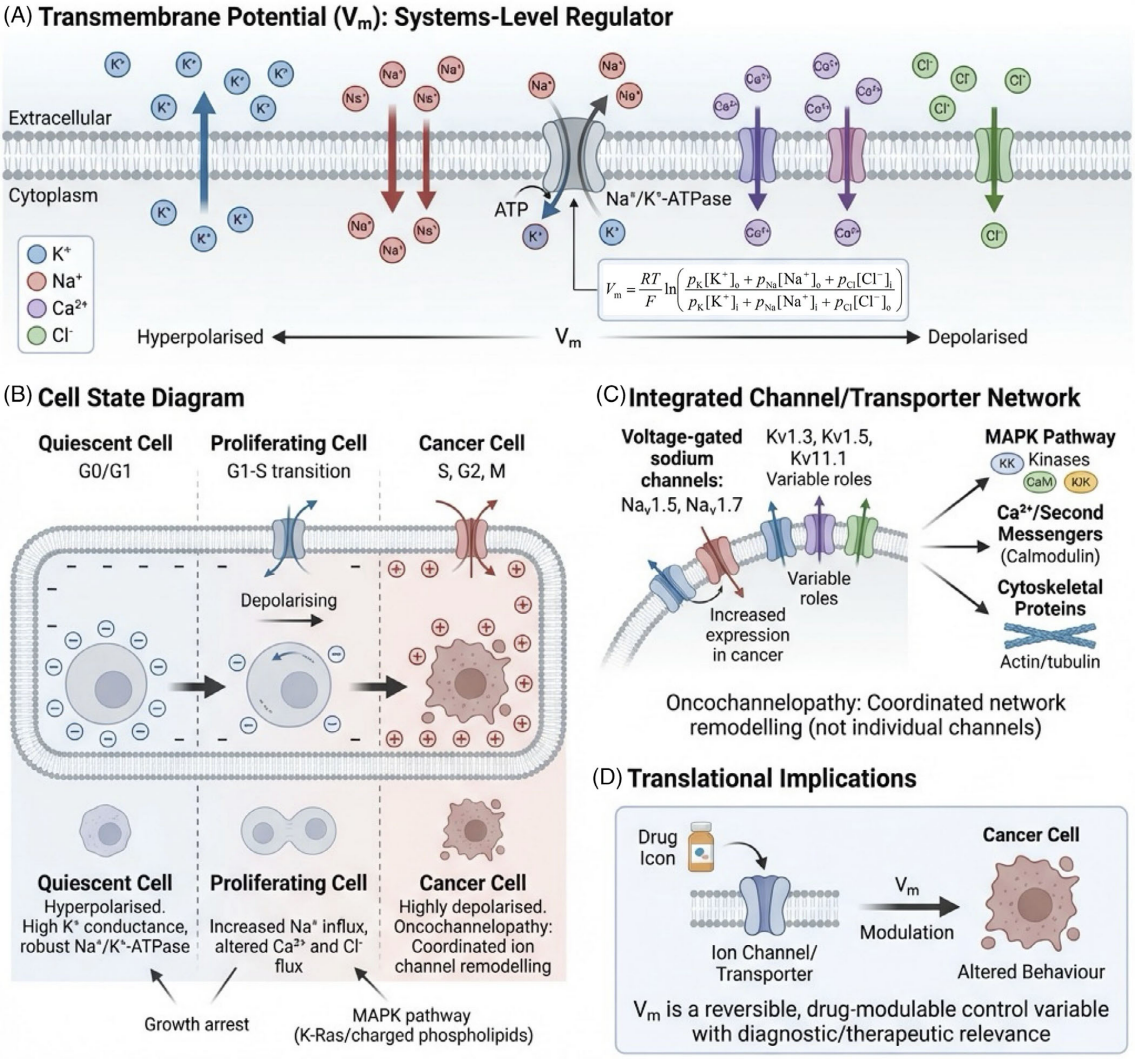
Systems‐level bioelectric regulation of cancer cell state. (A) The transmembrane potential (*V*_m_) emerges from the integrated activity of ion channels, transporters and electrogenic pumps. Potassium (K^+^), sodium (Na^+^), calcium (Ca^2^^+^) and chloride (Cl^−^) fluxes, together with the Na^+^/K^+^‐ATPase, establish electrochemical gradients that determine *V*_m_. The Goldman–Hodgkin–Katz framework illustrates how relative ion permeabilities and concentrations define the steady‐state membrane voltage. Shifts in ion conductance alter *V*_m_ along a continuum from hyperpolarised to depolarised states. (B) Conceptual cell‐state diagram linking *V*_m_ to functional phenotypes. Quiescent cells exhibit hyperpolarised membrane potentials maintained by dominant K^+^ conductance and pump activity. During cell‐cycle entry, depolarisation accompanies increased Na^+^ influx and altered Ca^2^^+^ and Cl^−^ dynamics. Cancer cells stabilise a persistently depolarised state associated with coordinated ion channel remodelling (‘oncochannelopathy’), supporting proliferation, plasticity and oncogenic signalling. (C) Integrated ion channel/transporter network in cancer. Voltage‐gated sodium channels (e.g., Na_v_1.5, Na_v_1.7), potassium channels (e.g., Kv1.3, Kv1.5, Kv11.1), and Ca^2^^+^/Cl^−^ channels form a coupled network that regulates *V*_m_ and downstream signalling. Depolarisation promotes activation of MAPK pathways, Ca^2^^+^‐dependent second messenger systems (e.g., calmodulin), and cytoskeletal remodelling. These effects arise from coordinated network behaviour, not single‐channel activity. (D) Translational implications. Membrane potential represents a reversible, drug‐modulable state variable that can be targeted pharmacologically, genetically or physically (e.g., ion channel modulators, optogenetics, tumour‐treating fields). Modulation of *V*_m_ alters cancer cell behaviour, including proliferation, differentiation and invasiveness, highlighting its potential as a diagnostic biomarker, pharmacodynamic readout and therapeutic control parameter.

Importantly, these effects cannot be understood at the level of individual channels alone. Cancer‐associated depolarisation arises from coordinated, network‐level changes in ion transport that integrate electrical, biochemical and mechanical processes. *V*_m_ therefore represents a systems‐level state variable that links ion‐channel activity with signalling, metabolism, and transitions in cell state (Figure 1).^23^, ^24^, ^45^

### 1.4 Aim and scope of this review

This review summarises around 15 years of research into the membrane potential in cancer, with the objective of establishing the regulation of the bioelectric state as an integrative and clinically actionable dimension of malignancy. Focusing beyond individual ion channels or specific tumour entities, we adopt a systems‐level approach, considering *V*_m_ as a state variable that connects membrane biophysics to signalling networks, metabolic regulation, and cell fate control.^13^, ^50^

To address current gaps, this review is organised into two complementary parts. The first part provides a quantitative and mechanistic synthesis of *V*_m_ across tumour systems, integrating evidence from model organisms, cell lines, and patient‐derived samples. The second part translates these insights into a structured roadmap for clinical implementation, including standardised *V*_m_ measurement, pharmacodynamic validation, and integration into precision oncology frameworks.

Our literature search adhered to the PRISMA 2020 guidelines and included English‐language studies published since 2010 examining membrane potential dynamics or *V*_m_‐targeted interventions in cancer models. Of the 358 records screened, 26 met the predefined inclusion criteria. These studies consistently associate depolarised *V*_m_ states with proliferation, stemness, therapy resistance, and invasive behaviour. This supports the central role of bioelectric state regulation in malignancy.

This review goes beyond descriptive accounts of cancer bioelectricity, integrating mechanistic evidence with translational considerations to inform experimental design, biomarker development, and therapeutic strategies. Crucially, it offers a unified framework combining: (i) a quantitative synthesis of *V*_m_ across tumours; (ii) systems‐level mechanistic integration of signalling, metabolism and cell‐state regulation; and (iii) a structured roadmap for clinical translation. By explicitly linking these three areas, the study elevates *V*_m_ from a descriptive parameter to a testable and actionable state variable in precision oncology.

## 2 LITERATURE RESEARCH AND TRANSLATION INTO ACTIONABLE DIRECTIONS

### 2.1 Positioning within existing literature and knowledge gaps

Over the past decade, an increasing number of reviews have emphasised the significance of bioelectric signals and ion channel dysregulation in cancer biology. Pioneering research has laid the groundwork for the concept of oncochannelopathies, which describes how the altered expression and function of ion channels can contribute to the processes of proliferation, migration and metastasis. Comprehensive reviews by Prevarskaya et al. and Litan and Langhans, in particular, have systematically linked potassium, sodium, calcium and chloride channels to key hallmarks of cancer, including uncontrolled proliferation and invasion.^23^, ^24^ In parallel, more recent reviews have expanded this perspective by highlighting the broader role of ion‐channel‐mediated bioelectric signalling in tumour progression, therapy resistance, and microenvironmental interactions.^50^, ^51^

Meanwhile, developmental and systems biology research has introduced bioelectricity as a fundamental regulator of cell fate, tissue patterning, and morphogenesis. Reviews by Levin and colleagues have been particularly influential in establishing the membrane potential as an instructive signal that can control large‐scale pattern formation and long‐range cellular communication.^1^, ^2^ Within this framework, *V*_m_ is understood not merely as a passive biophysical parameter, but as an active regulator of cellular decision‐making. More recent work has further reinforced this concept by linking bioelectric signalling to multiscale regulatory processes and disease states, including cancer.^50^, ^52^

More recently, emerging reviews have begun to reconcile these different approaches to cancer. For instance, Yang and Brackenbury emphasised the significance of membrane potential in cancer progression and proposed it as a potential therapeutic target,^3^, ^13^ while Payne et al. highlighted the importance of bioelectric control mechanisms in metastasis.^15^ These contributions represent important steps towards integrating bioelectricity into oncology and are complemented by recent efforts to position bioelectric signalling within translational and therapeutic frameworks.^50^, ^51^

However, despite these advances, the literature remains fragmented across three largely separate domains. Firstly, reviews focusing on cancer predominantly analyse individual ion channels or channel families without considering their collective impact on membrane potential at a systems level.^23^, ^24^ Secondly, reviews focusing on bioelectricity mainly address development and regeneration, with limited application to tumour biology or clinical oncology.^1^, ^2^ Thirdly, discussions of translation often highlight therapeutic opportunities but lack quantitative frameworks for *V*_m_ measurement, standardisation, and clinical implementation.^13^, ^15^

Consequently, membrane potential is often treated as a secondary parameter, not as a primary organising principle of cancer cell behaviour. This fragmentation limits both mechanistic understanding and translational progress.^3^, ^18^, ^23^

This review addresses these issues by adopting a systems‐level perspective, positioning *V*_m_ as an integrative state variable that links ion‐channel activity, signalling networks, metabolism and cell‐state transitions. Unlike prior reviews that primarily focus on individual ion channels or conceptual aspects of bioelectricity, this work introduces *V*_m_ as a quantitatively comparable, system‐level state variable and explicitly links mechanistic evidence to a structured translational framework.

By explicitly connecting mechanistic insights with clinical applicability, this work aims to advance the field from descriptive observations to a structured, testable bioelectric paradigm in cancer research. Remaining gaps include cross‐tumour quantitative *V*_m_ comparisons and clinical standardisation, as well as the development of predictive, attractor‐based, multiscale models that can link bioelectric state transitions to therapeutic decision‐making.^31^, ^32^, ^53^, ^54^, ^55^

### 2.2 Quantitative and mechanistic synthesis of *V*_m_ in cancer

In order to progress beyond descriptive observations, a quantitative, systems‐level synthesis of membrane potential across cancer models is required. Although individual studies have demonstrated *V*_m_ alterations in specific systems, a comparative framework integrating these findings across tumour types, measurement techniques and functional outcomes is lacking.^56^

In this section, we integrate experimental evidence from model organisms, cell lines and patient‐derived systems to identify common *V*_m_ patterns and their functional consequences. This approach enables us to identify conserved bioelectric features of malignancy and provides a foundation for quantitative comparisons and translational applications.

#### 2.2.1 *V*_m_ across tumour systems

Cancer cells consistently exhibit depolarised membrane potentials across diverse experimental systems, compared to their non‐transformed counterparts.^34^ This shift is observed across species, tumour types and measurement platforms, indicating that depolarisation is a conserved bioelectric hallmark of malignancy. In order to establish membrane potential as a measurable and comparable parameter across tumour systems, a quantitative synthesis is performed. Table 1 summarises the ranges of *V*_m_ reported, the experimental models used and the associated phenotypic states across cancer types. It provides a quantitative overview of *V*_m_ values across representative cancer models, including measurement approaches and associated functional outcomes.

**TABLE 1:** Quantitative overview of membrane potential (*V*_m_) across cancer systems.

Model system	Cell type	*V*_m_ (mV) cancer	*V*_m_ (mV) healthy/control	Measurement method	Functional association	Ref
*Xenopus laevis* (ITLS model)	Oncogene‐induced tumour‐like structures	∼ −10 to −30 *	∼ −50 to −70 *	Optical voltage imaging (voltage‐sensitive dyes)	Depolarisation precedes tumour formation and predicts ITLS development	^6^, ^12^
Human	Cancer stem cells (liver)	−7.0 ± 1.3 **	−23.0 ± 1.4 **	Microelectrode impalement	Depolarisation associated with stemness and tumourigenicity	^57^
Human glioblastoma	U87 CSC‐like cells	∼ −20 to −40 *	n/a	Patch‐clamp–based recordings	Depolarisation correlates with therapy resistance and quiescence	^5^
Human multiple myeloma	RPMI‐8226	−42 ± 2 **	n/a	Patch‐clamp (current‐clamp)	*V*_m_ oscillations regulate G1/S progression	^58^
Breast cancer	MDA‐MB‐231/MCF‐7	≈ −40 *	≈ −70 * (non‐transformed epithelial)	Voltage‐sensitive dyes	Depolarisation associated with increased proliferation	^59^
Breast cancer (population dynamics)	Multiple cell lines	−20 to −50 * (dynamic)	More hyperpolarised, stable *V*_m_	Optical voltage imaging (voltage‐sensitive dyes)	Electrical activity correlates with aggressive behaviour	^60^
Gastric cancer	MKN45	Variable (∼ −40 to −70 * AMPK‐dependent)	n/a	Patch clamp electrophysiology	Hyperpolarisation induces S‐phase arrest	^47^
Glioma (patient‐derived)	IDH‐WT glioma	∼ −20 to −50 * (variable)	n/a	Electrophysiology and calcium imaging	Neuron‐driven depolarisation enhances proliferation	^61^
Nav1.7‐expressing cells	Various cancer models	depolarised compared to controls *	More hyperpolarised controls *	Electrophysiology/functional assays	Sodium channel expression promotes depolarisation and invasive behaviour	^39^, ^41^, ^42^
Metastatic cancer cells	Multiple tumour types	−37 to −55 *	∼ −60 to −80 * More hyperpolarised	Mixed methods (patch clamp, voltage‐sensitive dyes)	Depolarisation correlates with metastatic potential	^15^

*Reported/approximated value or range (literature synthesis).

**Measured value (experimental).

The comparison reveals that cancer cells typically occupy a depolarised membrane potential range of approximately −10 to −50 mV. In contrast, differentiated or non‐proliferative cells are more commonly found in hyperpolarised states ranging from −50 to −90 mV. It is important to note that these values are not static; *V*_m_ exhibits dynamic fluctuations depending on cell‐cycle phase, metabolic state and microenvironmental context. However, direct quantitative comparisons between studies are limited by methodological differences, such as the use of patch‐clamp electrophysiology versus optical voltage imaging, and variations in calibration protocols.

Despite methodological variability the directionality of *V*_m_ changes is highly consistent. This suggests that depolarisation is not merely a model‐specific observation, but reflects a generalisable bioelectric feature of malignant transformation.^6^, ^12^

These findings support the interpretation of *V*_m_ as a quantitative state variable that captures functional differences between malignant and non‐malignant cells. Similar conclusions have also been reached in breast cancer models in which experimental alteration of the bioelectric state was sufficient to suppress malignant behaviour, further supporting *V*_m_ as a functional parameter, not merely a descriptive correlate.^12^, ^59^, ^60^ This provides a measurable and potentially actionable biomarker across cancer systems.

Although depolarisation is a common feature across many tumour systems, the regulation of membrane potential and the mechanisms by which it is established vary substantially between tumour types, molecular subtypes, and microenvironmental contexts, as do its functional consequences. This heterogeneity is evident in that solid tumours, such as those of the breast, brain and stomach, often exhibit pronounced depolarisation driven by coordinated ion‐channel remodelling. In contrast, haematological malignancies tend to rely more strongly on intracellular signalling and metabolic coupling, resulting in less well‐defined *V*_m_ signatures.^3^, ^5^, ^23^, ^24^, ^47^

In addition, tumour stage and differentiation state influence bioelectric profiles. Early‐stage tumours and proliferative cell populations tend to occupy dynamic *V*_m_ regimes associated with cell‐cycle progression. In contrast, advanced or therapy‐resistant tumours often stabilise depolarised states linked to stemness and plasticity.^5^, ^15^ These differences suggest that *V*_m_ should not be interpreted as a uniform marker, but as a context‐dependent parameter reflecting tumour heterogeneity.

Therefore, a systematic mapping of *V*_m_ distributions across tumour entities, molecular subtypes and disease stages is essential for translating bioelectric concepts into precision oncology. This would facilitate the identification of tumour‐specific bioelectric signatures and enhance patient stratification for *V*_m_‐targeted interventions.

#### 2.2.2 *V*_m_ as a regulator of proliferation and cell‐cycle progression

A central and consistently observed feature across cancer systems is the close relationship between membrane potential and proliferative capacity. *V*_m_ acts as a dynamic regulator of cell‐cycle progression and not merely as a passive correlate of cellular activity, with specific voltage states being associated with particular transitions in the cell cycle.^20^, ^22^, ^23^, ^58^

Proliferative cells occupy a depolarised *V*_m_ regime across multiple models, typically ranging from approximately −10 to −50 mV, whereas quiescent or differentiated cells exhibit more hyperpolarised states. Importantly, however, this relationship is not monotonic, but phase‐specific. Cell‐cycle progression is accompanied by reproducible *V*_m_ oscillations: hyperpolarisation is required for DNA synthesis during the S phase, while depolarisation facilitates transitions into the G1 phase and mitosis.^20^, ^21^, ^22^, ^25^ These findings suggest that *V*_m_ acts as a gating variable for cell‐cycle checkpoints, not merely indicating proliferation.^20^, ^21^, ^22^, ^25^

These voltage‐dependent effects mechanistically emerge from coordinated changes in ion‐channel activity and membrane organisation. Depolarisation has been shown to rearrange charged phospholipids within the plasma membrane, promoting the clustering of signalling molecules such as K‐Ras and activating downstream MAPK pathways.^36^ This establishes a direct link between membrane biophysics and canonical oncogenic signalling. Conversely, enforced hyperpolarisation, achieved through the pharmacological or genetic manipulation of ion conductances, induces reversible cell‐cycle arrest by inhibiting DNA synthesis and mitotic entry.^10^, ^12^

Further experimental perturbation studies demonstrate that the magnitude and timing of *V*_m_ changes are both critical. Moderate depolarisation is necessary for proliferation to begin, whereas excessive or prolonged depolarisation can disrupt the continuity of the cell cycle and induce arrest or cytotoxicity. For instance, blocking potassium channels with 4‐aminopyridine induces depolarisation and G1 arrest in multiple myeloma cells. Combining this with chemotherapeutic agents such as paclitaxel enhances the antiproliferative effects by disrupting phase‐specific *V*_m_ coordination.^58^, ^62^ These observations suggest that cancer cells rely on precisely regulated *V*_m_ trajectories, not fixed voltage states.^25^, ^48^, ^58^, ^62^

The tissue context may further influence these trajectories. In breast cancer, for example, left–right asymmetries in incidence and aggressiveness have been linked to corresponding epigenetic and bioelectric differences. These include altered methylation of hyperpolarising ion channel genes, as well as side‐dependent effects on membrane depolarisation and Ca^2^^+^ influx.^63^, ^64^, ^65^, ^66^ These findings suggest that the regional patterning of bioelectric control of proliferation may also exist at the tissue level.

In addition to ion‐channel‐mediated effects, membrane potential is closely linked to metabolic regulation. Activation of AMP‐activated protein kinase (AMPK) in gastric cancer cells induces membrane hyperpolarisation via modulation of chloride transport, resulting in S‐phase arrest and reduced proliferation.^17^, ^29^, ^47^ This coupling of energy status, ion transport and *V*_m_ provides further support for the interpretation of membrane potential as an integrative regulator that coordinates metabolic and proliferative decision‐making.

The membrane potential is also closely linked to metabolic regulation, forming an integrated bioelectric–metabolic network that supports tumour growth and adaptation. The activity of transporters involved in nutrient uptake, including glucose and amino acid transporters, is directly influenced by ion gradients maintained by *V*_m_.^17^, ^26^, ^29^, ^36^, ^67^ Conversely, metabolic pathways regulate ion channel activity and pump function, thereby shaping the bioelectric state.

Experimental evidence shows that metabolic sensors, such as AMP‐activated protein kinase (AMPK), can modulate *V*_m_ by regulating ion transport. This leads to hyperpolarisation and cell‐cycle arrest in gastric cancer cells.^47^, ^68^ Conversely, depolarised states are often associated with increased glycolytic activity and metabolic reprogramming, which supports proliferation and survival. Emerging studies further indicate that ion‐channel activity and *V*_m_ dynamics are tightly coupled to key metabolic pathways, including glycolysis and oxidative phosphorylation, linking bioelectric regulation to cellular energy homeostasis and redox balance.^17^, ^23^, ^29^, ^36^, ^69^

This bidirectional coupling suggests that *V*_m_ not only regulates cellular behaviour, but also reflects metabolic state.^67^, ^69^ Therefore, integrating bioelectric measurements with metabolic profiling could provide a more comprehensive understanding of tumour physiology and identify new vulnerabilities for therapeutic intervention. These findings establish *V*_m_ as a phase‐specific control parameter of cell‐cycle progression. Cancer cells exploit depolarised bioelectric states to sustain proliferation, yet they remain vulnerable to disturbances that disrupt the timing, amplitude or coordination of voltage transitions. This reveals bioelectric control points that can be targeted to selectively interfere with tumour growth.^22^, ^23^, ^50^, ^70^

#### 2.2.3 *V*_m_ in stemness, differentiation and cellular plasticity

Beyond its role in controlling the cell cycle, membrane potential is a key regulator of transitions in cell state, particularly the balance between stemness and differentiation. This is of critical relevance in cancer, where the persistence of cancer stem cell (CSC)‐like populations is a key driver of tumour progression, therapy resistance, and relapse.^14^, ^71^

Across multiple systems, CSCs consistently exhibit more depolarised membrane potentials than their non‐malignant or differentiated counterparts. For instance, CSCs derived from human liver tissue exhibit *V*_m_ values of around −7 mV, whereas normal stem cells display values of approximately −23 mV.^14^, ^57^, ^71^ This depolarised bioelectric state is closely associated with the maintenance of stemness, including the sustained expression of transcription factors such as OCT4, SOX2 and NANOG, and enhanced self‐renewal capacity.^14^, ^50^, ^71^

Experimental manipulation of *V*_m_ demonstrates a causal relationship between bioelectric state and differentiation potential. Forced hyperpolarisation, achieved through pharmacological, genetic or ionic interventions, promotes differentiation and reduces stem cell‐like properties in multiple cancer models. Conversely, depolarisation suppresses lineage commitment and stabilises undifferentiated states. Notably, even transient depolarisation during the early stages of differentiation can irreversibly inhibit lineage commitment, indicating that *V*_m_ acts at critical decision points in cell fate determination.^14^, ^50^, ^55^, ^70^, ^71^

Mechanistically, *V*_m_‐dependent control of stemness is mediated through multiple interconnected pathways. Changes in membrane potential regulate calcium influx and intracellular Ca^2^^+^ microdomains, influencing transcriptional regulators and epigenetic programmes that govern cell identity.^23^, ^29^, ^72^ In parallel, *V*_m_ modulates intracellular pH and metabolic fluxes via electrogenic transporters, thereby linking the bioelectric state to metabolic reprogramming. This concept is also consistent with the tumour‐suppressive role of transporters such as SLC5A8, which couple ion gradients to uptake of anti‐proliferative metabolites and thereby connect bioelectric state regulation with epigenetic control.^72^ These coupled processes establish *V*_m_ as an upstream regulator of cell‐state stability, not merely a consequence of differentiation.

Importantly, *V*_m_ defines a continuum of bioelectric states, not a binary switch.^1^, ^17^, ^31^, ^73^ CSCs occupy a depolarised regime that favours plasticity and adaptability, whereas hyperpolarised states promote differentiation, quiescence and reduced tumourigenic potential. This continuum model provides a framework for understanding how cancer cells transition between proliferative, stem cell‐like and differentiated states in response to internal and external stimuli.

In glioblastoma, for example, depolarised CSC‐like cells remain in a quiescent yet therapy‐resistant state, which is partly maintained by voltage‐gated sodium channel activity.^5^ Inhibiting these channels pharmacologically induces hyperpolarisation, promotes differentiation and reduces self‐renewal capacity. Channel‐dependent effects related to proliferation and Ca^2^^+^ handling have also been described in Ewing sarcoma. Aberrant KCNN1/SK1 expression contributes to the bioelectric control of malignant behaviour in this condition.^74^ This demonstrates that bioelectric modulation can reprogram malignant cell states without directly targeting oncogenic mutations.

In addition to the plasma membrane potential, the mitochondrial membrane potential (ΔΨm) is a key bioenergetic parameter that controls ATP production, reactive oxygen species (ROS) generation, and susceptibility to apoptosis. There is increasing evidence of functional coupling between *V*_m_, calcium signalling and ΔΨm in cancer cells. Membrane depolarisation can enhance the influx of Ca^2^^+^ into the cytosol, which in turn influences the uptake of calcium into the mitochondria and the stability of the ΔΨm, thereby linking the electrophysiology of the plasma membrane to metabolic reprogramming and cell survival pathways.^29^, ^47^, ^69^ Conversely, mitochondrial dysfunction and altered ΔΨm can affect ion channel activity and the cellular redox state, thereby shaping the global bioelectric profile. These findings suggest that bioelectric regulation in cancer involves interconnected membrane systems that integrate plasma membrane voltage with mitochondrial function and metabolic state and establish the membrane potential as a key regulator of cellular plasticity in cancer. By stabilising depolarised bioelectric states, tumours maintain stem cell‐like, therapy‐resistant populations. Conversely, targeted hyperpolarisation is a potential strategy for inducing differentiation, reducing plasticity and increasing therapeutic vulnerability.^17^, ^29^, ^36^, ^47^, ^50^

#### 2.2.4 *V*_m_ in migration and metastasis

In addition to its roles in proliferation and controlling the state of cells, the membrane potential is a key regulator of the migration of cancer cells and the dissemination of metastases. A growing body of evidence indicates that depolarised bioelectric states are associated with increased motility, invasiveness and organ‐specific colonisation across multiple tumour types.

Quantitatively, highly metastatic cancer cells typically exhibit depolarised *V*_m_ values in the range of approximately −37 to −55 mV. This regime overlaps with proliferative states, but is functionally distinct in its association with migratory behaviour.^15^ This suggests that *V*_m_ does not encode a single phenotype, but instead defines a multidimensional state space in which specific voltage regimes support distinct malignant functions.^15^, ^44^, ^55^, ^74^, ^75^

Mechanistically, *V*_m_ influences migration through several interconnected processes. One key pathway involves voltage‐gated sodium channels, which are frequently overexpressed in invasive cancers. Sodium influx through channels such as Nav1.5 and Nav1.7 contributes directly to membrane depolarisation and promotes cytoskeletal remodelling, extracellular matrix degradation and directional migration.^42^, ^44^, ^45^ These effects are partly mediated through coupling to intracellular pH regulation and protease activity, thereby linking bioelectric signals to the biochemical machinery of invasion.

In parallel, *V*_m_ modulates calcium dynamics, which play a central role in regulating the reorganisation of the actin cytoskeleton, the turnover of focal adhesions, and cell contractility. Depolarisation enhances calcium influx, activating downstream signalling pathways that facilitate migration and invasion.^23^, ^29^, ^44^, ^45^ Chloride channels also contribute to this process by regulating cell volume and osmotic balance, which are essential for movement through confined extracellular spaces. These ion fluxes form a coordinated bioelectric–mechanical system that enables metastatic behaviour.

Importantly, emerging evidence suggests that cancer cells can exploit spatial gradients in membrane potential to guide their migration in a specific direction. Bioelectric fields within tissues may provide instructive cues that influence cell movement, in a manner analogous to electrotaxis observed in developmental and wound‐healing contexts.^15^, ^31^, ^73^, ^76^ This raises the possibility that tumours utilise endogenous bioelectric landscapes to coordinate invasion and dissemination at the tissue level.

However, the relationship between *V*_m_ and metastasis is not strictly linear. Context‐dependent effects have been reported, such as hyperpolarisation suppressing proliferation but enhancing migratory capacity, and depolarisation reducing metastatic dissemination under certain conditions.^15^, ^44^, ^46^, ^74^, ^75^ These findings suggest that migration is influenced not only by absolute *V*_m_ values, but also by the interaction between ion fluxes, the cell cycle and the microenvironment.

In summary, the membrane potential acts as a regulator of metastatic competence, integrating bioelectric, mechanical, and biochemical processes. *V*_m_ defines a dynamic state space that enables cancer cells to transition between proliferative and migratory phenotypes, beyond simple promotion or inhibition of invasion. Targeting this bioelectric plasticity may therefore be a way to disrupt metastatic progression and limit tumour spread.

#### 2.2.5 *V*_m_ in multicellular and network‐level signalling

Although numerous studies have centred on *V*_m_ at the level of individual cells, emerging evidence suggests that cancer bioelectricity functions as a multicellular and network‐level phenomenon. Tumours are electrically coupled systems, not merely collections of independent cells, in which coordinated *V*_m_ dynamics can propagate across cell populations and influence collective behaviour.^1^, ^11^, ^12^, ^31^, ^33^, ^61^, ^77^ In addition to breast cancer and glioma, intrinsic electrical activity has also been associated with the progression of small‐cell lung cancer, providing further support for the idea that electrical signalling can drive malignancy in certain tumour types.^52^

High‐resolution voltage imaging studies have demonstrated that cancer cells exhibit temporally correlated dynamic *V*_m_ fluctuations across spatially separated cells.^60^ In breast cancer models, these fluctuations manifest as recurrent hyperpolarisation spikes and wave‐like propagation patterns, which are absent in non‐malignant epithelial cells.^60^ Such coordinated electrical activity suggests the presence of bioelectric communication networks, which are mediated by gap junctional coupling and local electric field interactions. Gap junctions, which are primarily formed by connexin proteins, provide direct cytoplasmic coupling between adjacent cells, enabling the propagation of electrical and ionic signals across tumour cell populations. Altered connexin expression and function have been widely reported in cancer and can disrupt intercellular communication, contributing to tumour progression, heterogeneity, and resistance.^22^, ^23^, ^24^ In this context, gap junctional coupling is a vital mechanism through which local changes in membrane potential can spread across tissues, facilitating coordinated bioelectric patterning at the multicellular level.^11^, ^12^

These network‐level dynamics provide a rapid, non‐diffusive mechanism for information transfer within tumours, complementing classical biochemical signalling pathways. Unlike paracrine or autocrine signalling, which rely on molecular diffusion, bioelectric signals can propagate over larger distances and in shorter timescales. This enables the synchronisation of cellular states, such as proliferation, migration and differentiation.^31^, ^32^, ^73^

In addition to tumour‐tumour communication, interactions between cancer cells and electrically active host tissues further expand the bioelectric network. In glioblastoma, for instance, tumour cells establish functional synaptic connections with neurons, enabling neuronal activity to depolarise cancer cells directly.^61^ This neuron‐to‐tumour signalling induces sustained depolarising currents and enhances tumour proliferation. Conversely, tumour cells can increase neuronal excitability through glutamatergic signalling, thereby establishing a bidirectional feedback loop that amplifies disease progression.^52^, ^61^, ^77^

At the tissue level, these interactions imply that tumours integrate into pre‐existing bioelectric circuits, hijacking physiological signalling networks to promote malignant growth. This reframes cancer progression as an emergent property of coupled cellular systems, not a purely cell‐autonomous process.^61^, ^77^

Importantly, network‐level bioelectricity also enables long‐range control of tumour behaviour. Experimental models have demonstrated that bioelectric perturbations in one region of tissue can influence tumour formation at distant sites, indicating that *V*_m_‐mediated signalling can operate across spatial scales beyond individual tumours.^3^, ^11^, ^12^ These findings are consistent with the concept of bioelectric fields acting as organising signals that coordinate cellular behaviour across tissues. This is in line with earlier hypotheses that linked membrane potential, sodium channels and gap junctions to growth regulation and tumour formation.^7^

These observations establish the membrane potential as a mediator of collective tumour behaviour, integrating single‐cell electrophysiology with tissue‐level organisation. *V*_m_ dynamics can synchronise malignant phenotypes, amplify signalling cascades and couple tumour cells to their microenvironment. This network perspective provides a conceptual framework for understanding how local bioelectric dysregulation can lead to systemic disease progression.^31^, ^32^, ^50^

From a translational perspective, targeting bioelectric coupling by modulating gap junctions, ion channels or applying external electric fields may disrupt tumour coordination and reduce collective behaviours such as invasion and resistance to therapy. Therefore, incorporating network‐level bioelectricity into experimental and computational models will be essential for capturing the full complexity of cancer as a bioelectric system.^23^, ^53^, ^78^, ^79^

#### 2.2.6 *V*_m_ in the tumour immune microenvironment

One of the key mechanisms through which *V*_m_ influences immune regulation is calcium signalling. The activation of T lymphocytes critically depends on sustained Ca^2^^+^ influx, driven by electrochemical gradients across the plasma membrane. Ion channels maintain the membrane potential, thereby regulating the driving force for Ca^2^^+^ entry through CRAC/Orai1 channels. Depolarising conditions can reduce this driving force, thereby impairing T cell activation, cytokine production and proliferation.^23^, ^29^, ^80^, ^81^

Notably, the tumour microenvironment is characterised by altered ionic conditions, including elevated extracellular potassium and sodium levels. These conditions can depolarise infiltrating immune cells and suppress their effector functions. These bioelectric alterations are consistent with the broader phenomenon of ion‐channel remodelling in cancer and its impact on cellular behaviour.^23^, ^24^, ^29^

Beyond T cells, ion‐channel‐mediated membrane potential regulation may also influence macrophage polarisation. Distinct ionic and metabolic states are associated with pro‐inflammatory (M1‐like) and anti‐inflammatory (M2‐like) phenotypes, suggesting that bioelectric signals may contribute to shaping tumour‐associated immune responses. However, direct causal evidence remains limited.^23^, ^29^, ^80^, ^81^

**FIGURE 2:**
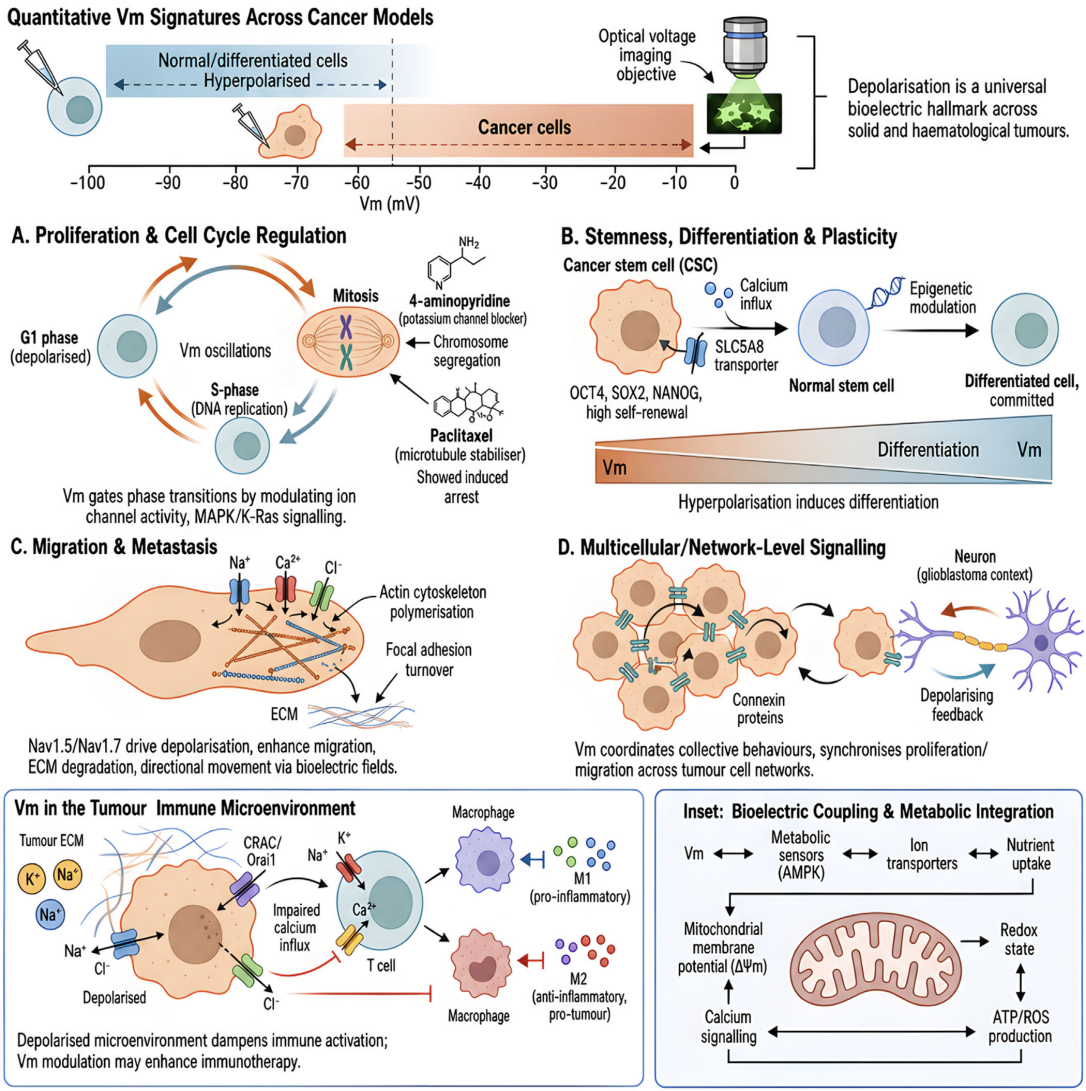
Membrane potential (*V*_m_) networks in cancer: quantitative and mechanistic overview. **Panel 1**. Quantitative *V*_m_ signatures across cancer models. Across experimental systems, malignant cells consistently exhibit depolarised membrane potentials relative to normal or differentiated cells. While non‐proliferative cells typically occupy hyperpolarised ranges (approximately −50 to −90 mV), cancer cells reside in more depolarised regimes (approximately −10 to −50 mV). These bioelectric states can be measured using electrophysiology or optical voltage imaging and represent a conserved, cross‐tumour hallmark of malignancy. **Panel 2**. Functional consequences of *V*_m_. (A) Proliferation and cell‐cycle regulation: *V*_m_ undergoes dynamic oscillations during cell‐cycle progression, with depolarisation facilitating G1 entry and mitosis, and hyperpolarisation supporting DNA synthesis during S phase. These voltage transitions regulate ion‐channel activity and signalling pathways such as MAPK/K‐Ras. Pharmacological perturbations (e.g., K^+^ channel blockers, microtubule stabilisers) disrupt *V*_m_ coordination and induce cell‐cycle arrest. (B) Stemness, differentiation and plasticity: Cancer stem cells (CSCs) exhibit depolarised *V*_m_ states associated with self‐renewal (e.g., OCT4, SOX2, NANOG expression). Hyperpolarisation promotes differentiation via Ca^2^^+^‐dependent signalling and epigenetic modulation, illustrating *V*_m_ as a regulator of cell‐state transitions. (C) Migration and metastasis: Depolarisation, driven in part by voltage‐gated sodium channels (Na_v_1.5/Na_v_1.7), enhances cytoskeletal remodelling, focal adhesion turnover and extracellular matrix (ECM) degradation. These processes support directional migration and invasive behaviour, potentially guided by bioelectric field gradients. (D) Multicellular and network‐level signalling: Tumours function as electrically coupled systems in which *V*_m_ dynamics propagate across cells via gap junctions (connexins) and local electric fields. In specific contexts, such as glioblastoma, neuronal activity directly depolarises tumour cells, establishing bidirectional signalling loops that promote proliferation and network synchronisation. **Panel 3**. *V*_m_ in the tumour immune microenvironment. Tumour cell depolarisation alters extracellular ionic conditions and electric fields, influencing immune cell function. Reduced Ca^2^^+^ influx in T cells (e.g., via CRAC/Orai1 channels) can impair activation, while *V*_m_‐dependent ionic and metabolic cues contribute to macrophage polarisation (M1 vs. M2 states). These effects suggest that bioelectric modulation may reshape the tumour–immune interface and enhance immunotherapeutic responses. **Inset**. Bioelectric coupling and metabolic integration. Membrane potential is tightly coupled to cellular metabolism. *V*_m_ regulates nutrient transport and ion flux, while metabolic sensors (e.g., AMPK) modulate ion channels and transporters. This bidirectional coupling links *V*_m_ to mitochondrial membrane potential (ΔΨm), calcium signalling, redox state and ATP/ROS production, establishing an integrated bioelectric–metabolic network in cancer.

From a translational perspective, these mechanisms imply that bioelectric modulation could enhance immunotherapy. Immune checkpoint pathways are closely linked to calcium signalling and metabolic state, and *V*_m_‐dependent modulation of Ca^2^^+^ dynamics may influence T cell activation and exhaustion. Therefore, modulating tumour or immune cell *V*_m_ could improve the efficacy of immune checkpoint inhibitors by restoring Ca^2^^+^‐dependent activation pathways and reversing tumour‐induced immunosuppression.^23^, ^29^, ^68^, ^80^ Figure 2 summarises the membrane potential (*V*_m_) networks in cancer.

### 2.3 Diagnostic and therapeutic exploitation of *V*_m_

The ability to measure and manipulate *V*_m_ sets bioelectricity apart from many other cancer‐relevant parameters, providing a direct link between mechanistic insight and clinical application. Unlike static molecular alterations, *V*_m_ is a dynamic, reversible and tuneable state variable that can function as a diagnostic biomarker, a pharmacodynamic readout and a therapeutic control target simultaneously.^13^, ^34^, ^50^, ^51^, ^60^, ^82^, ^83^

A large body of experimental research has examined the role of membrane potential in tumour development in various model systems. These studies provide a biological basis for understanding how *V*_m_ regulates cancer‐associated phenotypes. Table 2 summarises key studies examining *V*_m_ in tumour initiation, proliferation and differentiation, and includes comparisons with healthy cells.

**TABLE 2:** Overview of studies on membrane potential (*V*_m_) in tumour development, detailing investigated cell types, role of *V*_m_, and comparison with healthy cells.

Cell/tissue type	*V*_m_ involvement	Comparison with healthy cells	Ref
Oncogene‐induced tumour‐like structures in *Xenopus laevis*	Depolarisation of instructor cells gives rise to a metastatic phenotype of melanocytes	None provided	^6^
Oncogene‐induced tumour‐like structures in *Xenopus laevis*	Hyperpolarising the sites of tumour‐like structures inhibits their formation, while depolarisation resumes it	Sites of induced tumour‐like structures appear depolarised	^12^
Human multiple myeloma RPMI‐8226	Hyperpolarisation at the G1/S phase was shown to be necessary for progression through the cell cycle	None provided	^58^
Stem cells derived from human hepatocellular carcinoma	*V*_m_ differences are linked with differences in expression of GABA_A_ receptor subunits α_3_ and α_6_	Cancer stem cells are depolarised compared to normal ones	^57^
Breast cancer MDA‐MB‐231 and MCF7	Calcium influx needed for cell proliferation is driven by *V*_m_ depolarisation	Cancer cells are depolarised compared to healthy ones	^59^
Human gastric adenocarcinoma MKN45	ATP‐gated chloride channel CFTR causes hyperpolarisation when inhibited by increased AMPK, which stops the cell cycle in the S phase, while the depolarised cells continued proliferation	None provided	^47^
Patient‐derived glioma cells IDH‐WT	Driven by neuronal activity, glioma cells are depolarised and highly proliferative	None provided	^61^
Breast cancer MDA‐MB‐231	Left‐sided breast cancer cells show increased proliferation and depolarised *V*_m_ compared to right‐sided ones	None provided	^36^, ^63^
Breast cancer MDA‐MB‐231, MDAMB‐468, Cal‐51, SUM‐159, Hs578T, MDA‐MB‐453, and BT‐474, and T‐47D	Dynamic *V*_m_ fluctuations are associated with aggressive cell behaviour	*V*_m_ in cancer cells is significantly depolarised compared to healthy ones	^60^
Human glioblastoma U87	Chemotherapy resistance and stemness of cancer cells are correlated with a more depolarised *V*_m_	None provided	^5^
Breast cancer MCF‐7 and MDA‐MB‐231	Hyperpolarisation at the G1/S phase was shown to be necessary for progression through the cell cycle	None provided	^62^

These studies demonstrate that depolarised membrane potentials are consistently associated with proliferative, stem cell‐like and therapy‐resistant states. Conversely, hyperpolarisation is often associated with differentiation and growth suppression, suggesting that *V*_m_ plays a functional role beyond that of a purely descriptive parameter. These mechanistic insights have led to the exploration of a wide range of strategies to modulate membrane potential for therapeutic purposes.

Table 3 provides an overview of pharmacological, genetic and physical interventions targeting *V*_m_, together with the observed effects of these interventions on tumour behaviour and the reported selectivity of the interventions.

**TABLE 3:** Summary of studies on the pharmacological modulation of membrane potential in cancer treatment.

Cell/tissue type	Involvement of *V*_m_ in treatment	Reported effects	Selectivity and side effects	Ref
Ovarian cancer cells CP70 and A2780	***Pharmacological***: Application of positively charged gold nanoparticles resulted in depolarisation, which caused an increase in [Ca^2+^]_in_	No effect observed regarding proliferation or viability in tumour cells	Normal cell proliferation was halted, and cell death was induced	^84^
Human bladder cancer 253J	***Pharmacological***: Quercetin treatment of cancer cells induced hyperpolarisation by increasing K^+^ outward current	Cell viability reduced to 30.3 ± 13.5%	Not investigated in this study. Quercetin is generally well tolerated, with predominantly mild adverse effects reported^85^	^86^
Oncogene‐induced tumour‐like structures in *Xenopus laevis*	***Genetic***: Injecting mRNA encoding the ion channel Kir4.1 hyperpolarises cells	*V*_m_ hyperpolarisation prevents ITLS formation	No adverse effects were observed	^6^
Oncogene‐induced tumour‐like structures in *Xenopus laevis*	***Tumour marker***: Depolarisation is a reliable indicator for sites of ITLS formation before becoming histologically distinguishable	Not applicable	Average sensitivity of 48% and specificity of 81%	^12^
Oncogene‐induced tumour‐like structures in *Xenopus laevis*	***Genetic***: Embryos were injected with mRNA encoding the respective ion channel	Hyperpolarisation prevents ITLS formation at distant sites	No adverse effects were observed	^11^
Human multiple myeloma RPMI‐8226	***Pharmacological***: 4‐aminopyridine (4‐AP) was used to block voltage‐gated K^+^ channels and depolarise *V*_m_	Increased depolarisation caused mitotic arrest by preventing the G1/S transition in the cell cycle	Not investigated in this study. 4‐AP is an approved drug^62^ and was already successfully used in clinical trials for MS patients^87^	^58^
Human lung adenocarcinoma (A549) and non‐small‐cell lung cancer (patients FIS302 and FIS303)	***Pharmacological***: Tambjamine‐based compounds act as transmembrane anion transporters, causing hyperpolarisation	CSC differentiation attributed to *V*_m_ hyperpolarisation	Therapeutic doses were not toxic to healthy cells, while a reduction in the cancer cell population was observed	^14^
Rat pituitary tumour GH3	***Pharmacological***: An effective H_2_S concentration of > 4 µM activates specific K^+^ channels and hyperpolarises the tumour cells	Truncation of spontaneous action potentials and inhibition of exocytosis	Not investigated in this study. H_2_S is toxic even in small doses^88^	^89^
Induced tumour‐like structures in *Xenopus laevis* using *Kras^G12D^*	***Optogenetic***: Introducing archaerhodopsin (Arch) and channelrhodopsin‐2 (ChR2^D156A^) hyperpolarised cells at the site of injection	Hyperpolarisation inhibited growth of TLSs and a delayed activation of optogenetic channels reverted TLSs to normal tissue	Not investigated in this study. Other research reported that a certain degree of selectivity can be achieved by localised exposure to light^90^	^51^
Human melanoma (A2058), osteosarcoma (MG63, 143B, SAOS‐2, and HOS), neuroblastoma (NB‐1, and SK‐N‐SH), and murine osteosarcoma (LM8)	***Pharmacological***: *V*_m_ depolarisation in combination with TRAIL was found to be more effective than TRAIL alone	Cell death was attributed to mitochondrial network aberration	Not investigated in this study. TRAIL has already been reported to be highly selective in killing cancer cells^91^	^92^
Breast cancer MCF7 and MDA‐MB‐231	***Genetic***: Cells were transfected with a voltage‐gated Ca^2+^ channel (Cec). Cec is activated at around −40 mV but has defective inactivation.	Unregulated Ca^2+^ influx impedes cell proliferation and causes a caspase‐3‐mediated apoptosis	Given that healthy cells have a *V*_m_ much lower than the Cac activation threshold (see Table 2), they remained unaffected	^93^
Breast cancer MDA‐MB‐231, MDA‐MB‐468, and BT‐20	***Pharmacological***: K^+^ channel blocker (Amiodarone) hyperpolarises the cancer cells	Hyperpolarisation promotes and depolarisation suppresses metastasis and invasiveness	Not investigated in this study. Amiodarone is an already approved drug used to treat cardiac arrhythmias^94^, ^95^	^75^
Human cervical cancer cells HeLa	***Electromagnetic***: TTFs cause a change in *V*_m_	A correlation was observed between cell growth inhibition and changes in *V*_m_	Not investigated in this study. TTFs were reported to have minimal side effects^79^	^96^
Human melanoma SK‐Mel‐28	***Nanoparticles***: Nanoclays hyperpolarise *V*_m_	Hyperpolarisation may drive the antiproliferative effects of nano‐clays	Nano‐clays showed no toxicity to healthy cells	^90^
Rodent neuroblastoma NG108‐15 and human glioblastoma U87	***Pharmacological***: Using known ion‐channel agonists and antagonists causes changes in *V*_m_: NS1643, pantoprazole, retigabine, lamotrigine, and rapamycin	Phase in which the cell cycle was arrested varied with *V*_m_ changes, and hyperpolarisation promoted tumour differentiation	Toxicity to healthy neurons was demonstrated to be minimal. All compounds, with the exception of NS1643, are already approved for the treatment of other conditions	^70^
Breast cancer MCF‐7 and MDA‐MB‐231	***Pharmacological***: 4‐aminopyridine (4‐AP) in combination with Paclitaxel (PTX) was used to increase [K^+^]_in_ and depolarise *V*_m_	Cell‐cycle arrest is attributed to the depolarisation	Disrupts mitosis and shows enhanced antiproliferative effects in combination with ion‐channel modulation^58^, ^62^	^62^
Human glioblastoma U87	***Pharmacological***: Treatment with tetrodotoxin and QX‐314 blocked Na^+^ channels, causing hyperpolarisation	Stem cells differentiate and cease self‐renewal	Not investigated in this study. TTX is only mildly toxic in controlled doses and has already been successfully tested in cancer pain management trials^97^	^5^
Ewing sarcoma A‐673	***Genetic***: Silencing the *KCNN1* gene results in depolarisation	Depolarised *V*_m_ reduced Ca^2+^ entry and impeded cell division	Not investigated in this study	^74^

*Note*: It focuses on the types of cells and tissue investigated, the therapeutic interventions used, the tumour responses observed, and the treatment selectivity reported, along with reported side effects. Reported selectivity/side effects are limited to what was explicitly assessed in the cited study (or clearly labelled as prior clinical knowledge when applicable).

These studies demonstrate that membrane potential is a measurable biomarker and a controllable variable. However, the effects of *V*_m_ modulation are highly context‐dependent, emphasising the importance of state‐aware therapeutic strategies that consider tumour type, cell‐cycle phase and microenvironmental interactions.

#### 2.3.1 *V*_m_ as a diagnostic and functional biomarker

A key translational opportunity lies in using *V*_m_ as a functional biomarker of the malignant state. Experimental studies have shown that depolarisation can precede detectable morphological transformation and predict tumour formation with a level of performance comparable to that of established molecular markers in controlled models.^6^, ^12^, ^60^ This suggests that *V*_m_ may provide an early functional readout of tumourigenic state transitions.

Advances in voltage‐sensitive dyes, genetically encoded voltage indicators and fluorescence lifetime imaging now enable the high‐resolution, quantitative mapping of *V*_m_ in live cells and tissues.^60^, ^83^, ^98^, ^99^, ^100^ These approaches allow discrimination between malignant and non‐malignant cells based on their electrical signatures, enabling the detection of dynamic *V*_m_ fluctuations associated with aggressive behaviour.

In a translational context, integrating *V*_m_ measurements into ex vivo biopsy workflows could provide rapid orthogonal information alongside histopathology and genomic profiling. Intraoperative *V*_m_ mapping could further improve tumour margin assessment, while longitudinal *V*_m_ monitoring could serve as a pharmacodynamic biomarker to evaluate treatment response in real time. However, achieving clinical applicability will require the standardisation of calibration protocols, dye performance and measurement conditions to ensure reproducibility and comparability across studies.^60^, ^83^, ^98^, ^100^

#### 2.3.2 Pharmacological and genetic modulation of *V*_m_

Pharmacological modulation of *V*_m_ is a versatile strategy for reshaping malignant bioelectric states. A variety of ion channel modulators, such as potassium channel activators, sodium channel blockers and synthetic anion transporters, have been demonstrated to affect the proliferation, differentiation and apoptosis of cancer cells.^5^, ^14^, ^38^, ^70^, ^74^, ^86^, ^89^, ^101^ In many cases, enforced hyperpolarisation suppresses proliferation and promotes differentiation, particularly in cancer stem cell‐like populations.

Importantly, *V*_m_ modulation can also act as a sensitisation strategy. Controlled depolarisation has been shown to increase susceptibility to agents that induce apoptosis, such as TRAIL, by altering mitochondrial function and calcium dynamics.^92^ These findings suggest that bioelectric interventions could complement existing therapies by rendering tumour cells more susceptible to treatment. Studies showing that depolarisation can potentiate TRAIL‐induced apoptosis also support this sensitisation concept, thereby reinforcing its therapeutic relevance.^91^, ^102^

Genetic approaches provide additional specificity and mechanistic insight. Optogenetic tools enable the precise and reversible control of *V*_m_ via light‐driven ion transport, facilitating the direct investigation of the causal relationships between membrane potential and tumour behaviour.^51^ Similarly, engineered ion channels can selectively target cancer cells based on their bioelectric properties, inducing apoptosis or growth arrest without affecting surrounding healthy tissue.^93^ These strategies highlight the potential for highly controlled, spatially restricted bioelectric interventions. In addition, repurposing clinically established ion‐channel drugs, including antiarrhythmics, alongside broader *V*_m_‐modulating strategies targeting potassium, sodium and calcium channels remains an attractive route for translation, although potential electrophysiological side effects must be carefully considered.^23^, ^24^, ^33^, ^38^, ^46^, ^50^, ^94^, ^95^, ^103^

The translational interpretation of several *V*_m_‐modulating compounds also depends on existing pharmacological and safety knowledge from non‐oncology contexts, including antiarrhythmics, 4‐aminopyridine, paclitaxel and hydrogen sulphide donors.^70^, ^87^, ^88^, ^94^, ^95^, ^104^, ^105^ Toxicity considerations for compounds such as hydrogen sulphide donors further illustrate the importance of dose‐dependent safety evaluation.^88^

#### 2.3.3 Physical and bioelectronic interventions

Physical approaches offer clinically scalable methods of modulating *V*_m_ that do not rely on systemic pharmacology. Tumour‐treating fields (TTFields) are the most advanced example and have an established clinical use in the treatment of glioblastoma and mesothelioma.^78^, ^79^, ^82^, ^106^ Although their mechanisms are still being investigated, accumulating evidence suggests that TTFields disrupt membrane potential dynamics and ion‐channel activity, thereby contributing to anti‐proliferative and pro‐apoptotic effects.^78^, ^79^, ^96^, ^106^, ^107^

Theoretical and experimental studies suggest that cancer cells may experience greater *V*_m_ fluctuations under alternating electric fields due to variations in membrane properties, size, and dielectric characteristics. This differential sensitivity may contribute to the tumour selectivity observed with TTFields, suggesting that bioelectric vulnerabilities can be exploited through externally applied fields.^98^, ^101^, ^106^

Alongside electromagnetic approaches, charged nanomaterials and electroactive compounds have emerged as tools for manipulating membrane potential and cellular behaviour. These materials interact with the plasma membrane via electrostatic mechanisms, thereby influencing ion flux, intracellular signalling and drug uptake.^84^, ^90^ While the exact contribution of *V*_m_ modulation versus secondary effects is yet to be fully resolved, these methods demonstrate the wider potential of bioelectric engineering in oncology.^84^, ^90^, ^108^, ^109^

#### 2.3.4 Towards *V*_m_‐guided precision oncology

Despite promising experimental and early translational results, there are several challenges that must be addressed before *V*_m_ can be fully integrated into clinical oncology. One major limitation is the lack of standardised, high‐throughput methods for measuring membrane potential in patient samples. Although patch‐clamp electrophysiology is still considered the gold standard, it is not easily scalable, so robust optical or indirect measurement techniques that are suitable for clinical workflows must be developed.^83^, ^98^, ^100^

Another key challenge is selectivity. As ion channels and transporters are vital for healthy tissues, particularly in the heart and nervous system, *V*_m_‐targeted interventions must be tumour‐specific to minimise off‐target effects. Exploiting cancer‐specific ion channel expression patterns or bioelectric vulnerabilities may enable selective targeting.

It is important to note that *V*_m_ should not be viewed as a standalone target, but as an additional axis within existing precision oncology frameworks.^13^, ^50^, ^110^, ^111^, ^112^ Combining bioelectric state information with genomic, transcriptomic and metabolic data could allow for more precise patient classification and optimisation of therapy. In this context, *V*_m_ can serve as a functional layer that captures the integrated output of multiple molecular perturbations.

Additionally, computational modelling approaches and emerging digital twin frameworks offer powerful tools for simulating *V*_m_ dynamics, predicting responses to bioelectric interventions, and optimising therapy in silico.^48^, ^53^, ^54^, ^56^, ^70^, ^111^, ^112^, ^113^, ^114^ These approaches build on earlier bioelectric simulation platforms that model the emergence of voltage patterns, voltage‐mediated signalling, and their impact on tissue‐level organisation, thereby linking single‐cell electrophysiology to multicellular behaviour across spatial and temporal scales.^31^, ^32^ These frameworks enable hypothesis testing in silico and represent an important step towards predictive, multiscale modelling of bioelectric regulation in cancer.

These considerations establish membrane potential as a clinically actionable parameter with significant diagnostic and therapeutic potential. By enabling the direct measurement and manipulation of the cellular state, bioelectric approaches offer a complementary strategy to molecular targeting. This has the potential to enhance treatment efficacy, overcome resistance and improve patient outcomes.

#### 2.3.5 Translational gap between preclinical findings and clinical implementation

Despite strong experimental evidence supporting the role of voltage‐gated ion channels in tumour biology, the translation of bioelectric interventions into clinical practice remains limited.^78^, ^109^, ^110^ With the exception of tumour‐treating fields, most *V*_m_‐modulating strategies remain confined to preclinical models, including in vitro systems and non‐mammalian organisms such as Xenopus laevis.^6^, ^11^, ^12^, ^101^

Several factors contribute to this translational gap. Firstly, many experimental studies rely on simplified systems that do not fully capture the complexity of the human tumour microenvironment. Secondly, the systemic effects of ion channel modulation raise safety concerns and off‐target toxicity, particularly in excitable tissues such as the heart and nervous system. Thirdly, the lack of standardised protocols for measuring *V*_m_ in patient samples complicates the integration of bioelectric biomarkers into clinical workflows.^17^, ^23^, ^50^, ^78^

**FIGURE 3:**
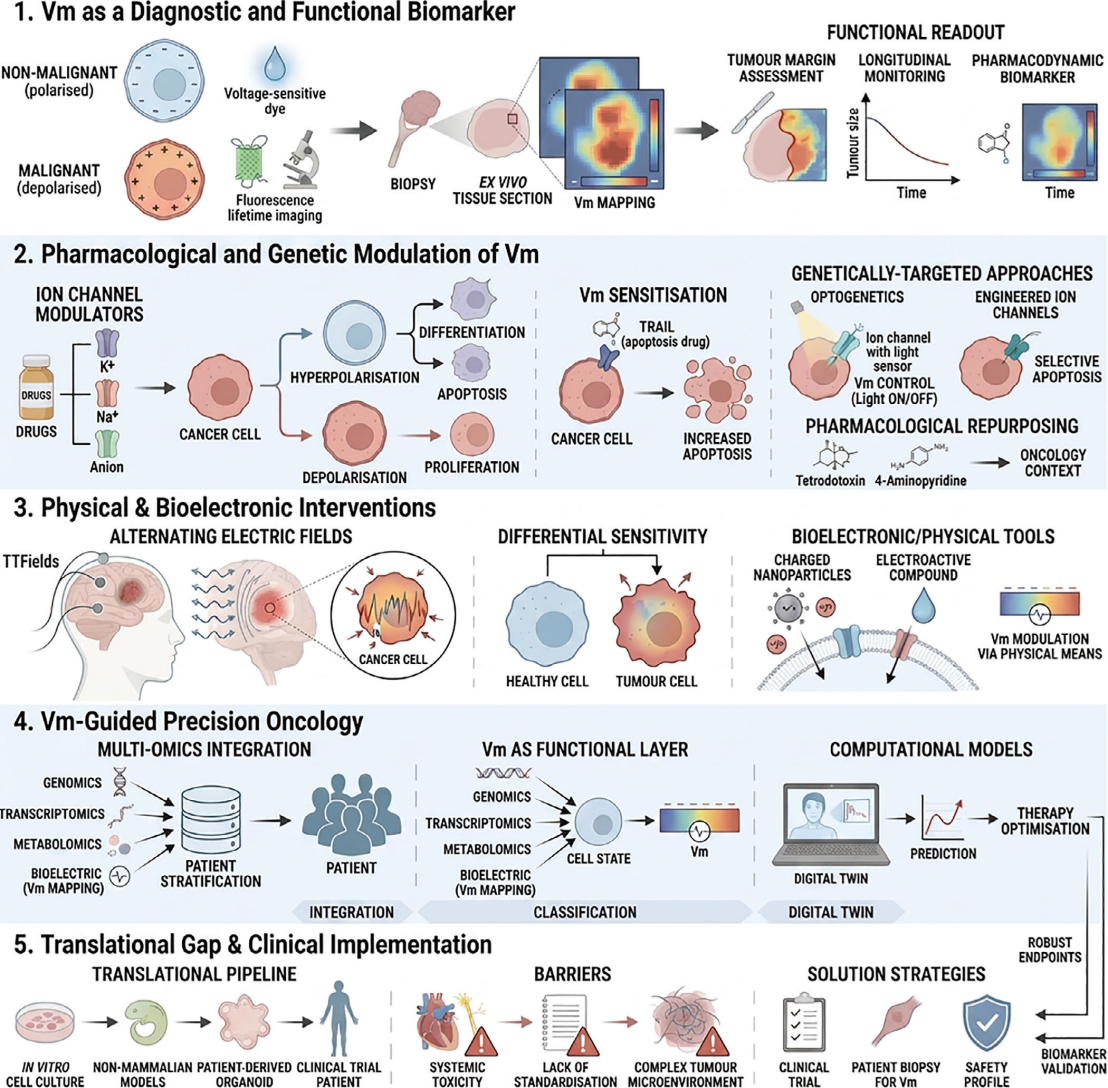
Translational exploitation of membrane potential (*V*_m_) in cancer: from biomarker to precision oncology. **(1)** *V*_m_ as a diagnostic and functional biomarker: Malignant cells exhibit depolarised membrane potentials relative to non‐malignant cells, enabling discrimination using voltage‐sensitive dyes and fluorescence lifetime imaging. Quantitative *V*_m_ mapping in biopsy‐derived tissues provides functional information for tumour margin assessment, longitudinal monitoring of tumour dynamics, and pharmacodynamic evaluation of treatment response. **(2)** Pharmacological and genetic modulation of *V*_m_: Ion channel modulators targeting K^+^, Na^+^ and anion conductances reshape bioelectric states, with hyperpolarisation generally promoting differentiation or apoptosis and depolarisation supporting proliferation. *V*_m_ modulation can also sensitise tumour cells to therapies (e.g., TRAIL‐induced apoptosis). Genetic approaches, including optogenetics and engineered ion channels, enable precise and reversible control of *V*_m_, while repurposing established ion‐channel drugs (e.g., tetrodotoxin, 4‐aminopyridine) represents a translational strategy. **(3)** Physical and bioelectronic interventions: Non‐invasive approaches such as tumour‐treating fields (TTFields) and other alternating electric field modalities exploit differential bioelectric properties of cancer cells to disrupt proliferation. Additional strategies include charged nanoparticles and electroactive compounds that modulate membrane potential via electrostatic interactions, highlighting scalable bioelectronic approaches for *V*_m_ control.^84^, ^90^, ^108^ **(4)** *V*_m_‐guided precision oncology: Integration of *V*_m_ measurements with multi‐omics data (genomics, transcriptomics, metabolomics) enables patient stratification and defines *V*_m_ as a functional layer linking molecular alterations to cell state. Computational models and digital twin approaches can simulate bioelectric dynamics, predict treatment responses and optimise therapy strategies in silico. **(5)** Translational gap and clinical implementation: Despite strong preclinical evidence, clinical translation remains limited due to challenges including systemic toxicity, lack of standardised *V*_m_ measurement protocols, and tumour microenvironment complexity. Bridging this gap requires validation in clinically relevant models, incorporation of *V*_m_ as a pharmacodynamic endpoint in trials, and development of standardised measurement and safety frameworks.

Bridging this gap will require coordinated efforts to validate bioelectric targets in clinically relevant models, such as patient‐derived organoids and in vivo systems, as well as establishing robust safety profiles for *V*_m_‐modulating interventions. Additionally, early‐phase clinical trials should incorporate *V*_m_ measurements as pharmacodynamic endpoints to evaluate target engagement and treatment response. Such strategies will be essential for translating promising preclinical findings into clinically actionable therapies (see Figure 3).

### 2.4 Translational roadmap: from bioelectric mechanisms to clinical implementation

**FIGURE 4:**
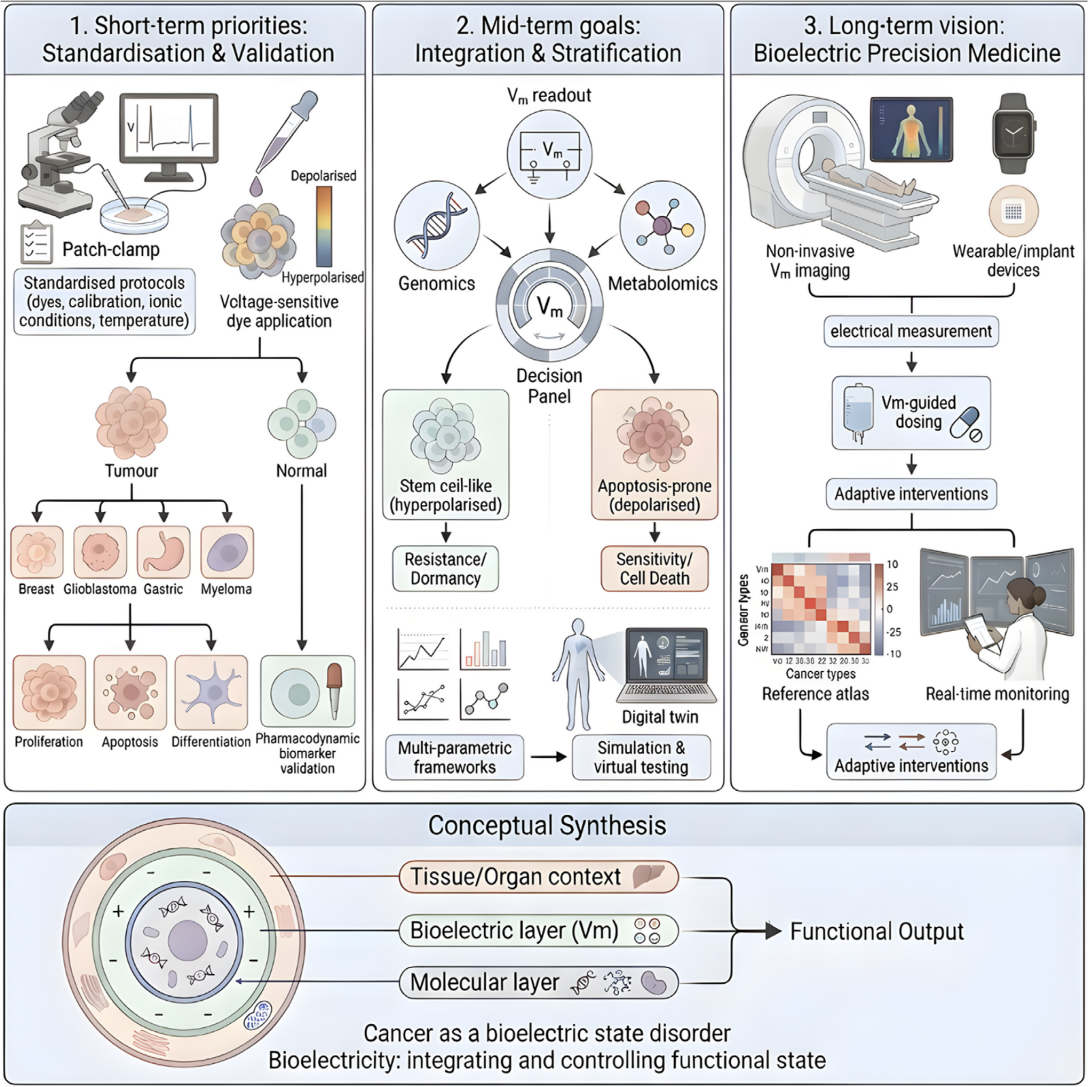
Translational roadmap: from bioelectric mechanisms to oncology practice. Stepwise framework for integrating *V*_m_ into cancer research and clinical application, progressing from standardisation to bioelectric precision medicine. **(1)** Short‐term priorities: standardisation and validation: The initial step focuses on establishing robust and reproducible methods for *V*_m_ measurement, including patch‐clamp electrophysiology and optical approaches using voltage‐sensitive dyes. Standardisation of calibration protocols, ionic conditions and experimental parameters is essential to enable quantitative comparisons across studies. In parallel, *V*_m_ must be validated as a functional biomarker by linking bioelectric states to phenotypic outcomes such as proliferation, apoptosis and differentiation across tumour types. **(2)** Mid‐term goals: integration and stratification: In the intermediate phase, *V*_m_ is incorporated into multi‐parameter frameworks alongside genomic, transcriptomic and metabolomic data to improve patient stratification. *V*_m_ functions as a dynamic, integrative layer reflecting cellular state (e.g., stem cell‐like, proliferative or apoptosis‐prone). Computational modelling and digital twin approaches enable simulation of bioelectric dynamics, prediction of therapeutic responses and virtual testing of intervention strategies. **(3)** Long‐term vision: bioelectric precision medicine: Future applications include non‐invasive *V*_m_ imaging, wearable or implantable bioelectronic devices and adaptive treatment strategies guided by real‐time bioelectric feedback. *V*_m_‐guided dosing and intervention aim to shift tumour cells between functional states, while reference atlases of tumour‐specific bioelectric profiles support personalised treatment decisions. **Conceptual synthesis**: Membrane potential represents a distinct regulatory layer that integrates molecular signals with tissue‐level context to determine functional cellular output. Framing cancer as a disorder of dysregulated bioelectric state control highlights *V*_m_ as both a measurable biomarker and a manipulable control variable, providing a complementary axis for precision oncology.^2^, ^12^

The evidence integrated in this review supports the interpretation of membrane potential as an integrative and clinically actionable regulator of cancer biology. However, translating this concept into clinical practice requires a structured framework that connects mechanistic understanding, quantitative measurement and therapeutic application. Here, we present a stepwise translational roadmap that positions the control of the bioelectric state as a complementary axis within precision oncology (Figure 4).

#### 2.4.1 Short‐term priorities: standardisation and validation

The immediate priority is to establish robust, standardised methods for measuring *V*_m_ in experimental and clinical settings.^60^, ^83^, ^98^ This includes the harmonisation of voltage‐sensitive dyes, calibration protocols, ionic conditions and temperature control, in order to ensure reproducibility and comparability across laboratories.^83^, ^99^

In parallel, *V*_m_ must be validated as a pharmacodynamic (PD) biomarker by systematically linking voltage changes to functional outcomes such as proliferation, apoptosis, and differentiation. Incorporating *V*_m_ measurements into preclinical studies and early‐phase clinical trials will be critical for establishing its predictive and monitoring value.^6^, ^10^, ^14^, ^50^, ^51^, ^58^, ^110^

Near‐term studies should prioritise tumour entities with existing quantitative *V*_m_ evidence, such as breast cancer, glioblastoma, gastric cancer and myeloma, using paired tumour‐versus‐normal biopsy profiling, standardised optical *V*_m_ calibration, and prospective pharmacodynamic monitoring in early‐phase intervention studies.^47^, ^58^, ^59^, ^60^, ^83^

#### 2.4.2 Mid‐term goals: integration and stratification

In the medium term, *V*_m_ measurements should be incorporated into multi‐parameter precision oncology frameworks alongside genomic, transcriptomic and metabolic profiling. This will enable *V*_m_ to serve as a functional readout capturing the combined effects of multiple molecular alterations.^1^, ^4^, ^23^, ^25^, ^50^, ^51^, ^110^

Biomarker‐guided therapeutic strategies can then be developed by aligning *V*_m_‐modulating interventions with specific tumour states. For instance, hyperpolarising approaches could be employed to promote differentiation in stem cell‐like tumours, while controlled depolarisation might increase susceptibility to apoptosis‐inducing therapies.^14^, ^51^, ^67^, ^92^, ^102^

Computational modelling and digital twin approaches will play a central role at this stage. Existing bioelectric models can simulate *V*_m_ dynamics, ion‐channel perturbations and treatment responses. This supports hypothesis testing and the virtual screening of therapeutic strategies.^48^, ^55^, ^111^, ^112^, ^115^

However, the creation of fully personalised digital twins is a long‐term goal that requires advances in parameter identifiability, multimodal data integration, and the development of rigorous verification, validation, and uncertainty quantification (VVUQ) frameworks.^31^, ^48^, ^53^, ^54^, ^111^, ^112^, ^113^, ^114^

#### 2.4.3 Long‐term vision: bioelectric precision medicine

In the long term, advances in bioelectric measurement and control could make fully integrated bioelectric precision medicine possible.^115^ This includes developing non‐invasive *V*_m_ imaging technologies, implantable or wearable electroceutical devices and *V*_m_‐guided dosing protocols that can adjust therapy dynamically based on real‐time bioelectric feedback.^50^, ^51^, ^54^, ^60^, ^78^, ^83^, ^110^

Such approaches could enable clinicians to actively influence tumour states by moving cells away from pathological bioelectric attractors and towards less aggressive or more therapy‐sensitive states. In this context, *V*_m_ would function not only as a biomarker, but also as a control variable within adaptive treatment strategies.^2^, ^100^, ^111^, ^116^

Another key objective is to develop reference atlases mapping *V*_m_ distributions across tumour types, stages and microenvironmental contexts. These atlases would provide a baseline for identifying tumour‐specific bioelectric signatures and enable comparative analyses across patient cohorts.^3^, ^4^, ^50^, ^60^, ^83^, ^110^

#### 2.4.4 Conceptual synthesis: cancer as a bioelectric state disorder

This roadmap supports a conceptual shift in cancer biology. We propose that malignancy can be conceptualised as a disorder of dysregulated bioelectric state control within a broader multi‐layered regulatory framework, consistent with emerging hypotheses in other disease domains where restoration of membrane potential is proposed as a therapeutic strategy.^2^, ^12^, ^34^, ^50^, ^55^ In this model, membrane potential integrates and constrains multiple layers of cellular regulation. More broadly, this view aligns with the idea that bioelectricity is a universal and multifaceted signalling mechanism operating throughout development, regeneration and disease.^73^, ^117^

This perspective does not replace existing frameworks, but complements them by introducing a physically grounded, measurable and manipulable variable that links molecular perturbations to functional outcomes and aligns with emerging perspectives advocating the integration of novel regulatory dimensions into next‐generation cancer therapies.^4^, ^12^, ^13^, ^110^, ^118^ Importantly, bioelectric interventions do not compete with established therapies, but provide an additional axis of control that can enhance the precision, timing and efficacy of these therapies.

By incorporating membrane potential into the conceptual and clinical framework of oncology, this approach aims to extend the scope of precision medicine from static molecular profiles to dynamic, state‐based intervention strategies.^54^, ^111^

### 2.5 Limitations of current bioelectric cancer research

Despite substantial progress, the current body of research on cancer bioelectricity has several important limitations that must be considered when interpreting its potential for translation into clinical practice.

One major limitation is the heavy reliance on in vitro systems and non‐mammalian models, such as Xenopus laevis, that may not accurately reflect the complexity of human tumours and their microenvironment.^11^ While these models provide valuable mechanistic insights, their predictive value for clinical outcomes is uncertain.

In addition, methodological variability in *V*_m_ measurement presents a significant challenge. Differences in electrophysiological techniques, voltage‐sensitive dyes, calibration protocols, and experimental conditions complicate quantitative comparisons across studies and limit reproducibility.^60^, ^83^, ^98^

Another limitation is the incomplete integration of bioelectric signalling with other regulatory layers, including genetics, epigenetics, metabolism, and immunity. Although there is increasing evidence to support interactions between these domains, a unified systems‐level framework has yet to be established.^23^, ^29^, ^31^, ^69^, ^81^

Finally, clinical validation of *V*_m_ as a biomarker or therapeutic target is still in its infancy. Large‐scale studies demonstrating its predictive value, specificity and safety in patients are lacking. This raises the possibility that current findings may overestimate the applicability of bioelectric interventions in clinical practice.^51^, ^78^, ^110^ Addressing these limitations is essential for advancing the field from experimental observations to clinically relevant applications.

## 3 CONCLUSION

This review summarises the growing body of evidence suggesting that the membrane potential is a key regulator of cancer cell behaviour. By integrating findings from ion channel biology, bioelectricity and oncology, we demonstrate that *V*_m_ is an active determinant of proliferation, differentiation, migration and therapy response, not merely a downstream consequence of molecular alterations.^3^, ^13^, ^15^, ^23^, ^34^

A key contribution of this work is shifting the focus from a reductionist, channel‐centric perspective to a systems‐level understanding of bioelectric regulation. By framing *V*_m_ as an integrative state variable, we offer a unifying concept that links ion‐channel activity to signalling pathways, metabolic states and phenotypic plasticity. Quantitative synthesis of *V*_m_ across tumour types further highlights that bioelectric states are measurable, comparable and potentially exploitable for clinical purposes.^59^, ^60^, ^83^

Despite significant advances, major gaps remain. Current studies are limited by methodological variability, a lack of standardised measurement protocols, and insufficient integration into clinical research pipelines. Overcoming these challenges is essential to establishing *V*_m_ as a reliable biomarker and therapeutic target.^51^, ^110^

Looking to the future, integrating bioelectric parameters into precision oncology frameworks promises to offer a new dimension to cancer diagnosis and treatment. Future strategies may seek to modulate global bioelectric states, thereby influencing entire regulatory networks simultaneously, without focusing exclusively on individual molecular targets. In this context, *V*_m_ could function as both a diagnostic indicator and a controllable variable for therapeutic intervention. This transition may be further accelerated by computational modelling and digital twin approaches, which enable the in silico predictive, state‐based optimisation of bioelectric therapies.^53^, ^54^, ^111^, ^112^, ^113^, ^114^

Based on the available evidence, cancer cannot be reduced to a purely bioelectric disease. However, bioelectric signalling emerges as a fundamental and previously underappreciated organising dimension of tumour biology, interacting with genetic, epigenetic, and metabolic regulatory layers to shape malignant cell states.

In conclusion, we propose that cancer can be understood as a disorder involving the dysregulation of bioelectric state control. Adopting this viewpoint could open up new avenues for research and clinical applications, potentially complementing existing molecular approaches and advancing the development of dynamic, state‐based precision oncology.

## AUTHOR CONTRIBUTIONS

C.D. reviewed the literature, and wrote, drafted, and edited the manuscript. R.S. conducted the literature search and drafted selected sections of the manuscript. S.G., D.G., M.K. and N.K. reviewed and edited the manuscript. C.A.E.H. and D.B. reviewed the literature and contributed to manuscript editing. C.B. conceptualised the study, contributed to writing and editing, and approved the final version of the manuscript.

## CONFLICT OF INTEREST STATEMENT

The authors declare that they have no competing interests.

## ETHICS STATEMENT

Not applicable.
